# Astaxanthin Improved the Quality of Hu Ram Semen by Increasing the Antioxidant Capacity and Mitochondrial Potential and Mitigating Free Radicals-Induced Oxidative Damage

**DOI:** 10.3390/ani14020319

**Published:** 2024-01-19

**Authors:** Tariq Sohail, Liuming Zhang, Xuyang Wang, Caiyu Jiang, Jian Wang, Xiaomei Sun, Yongjun Li

**Affiliations:** Key Laboratory for Animal Genetics & Molecular Breeding of Jiangsu Province, College of Animal Science and Technology, Yangzhou University, Yangzhou 225009, China; drtariqsohail34@yahoo.com (T.S.); 18352767281@163.com (L.Z.); 18705271593@163.com (X.W.); 19352674787@163.com (C.J.); jianwang1223@163.com (J.W.); xiaomeisun_yz@163.com (X.S.)

**Keywords:** Hu ram semen, 4 °C preservation, astaxanthin, sperm quality, antioxidant capacity

## Abstract

**Simple Summary:**

Sheep’s sperm is very sensitive to free radical-mediated oxidative and per oxidative damage during preservation at 4 °C, leading to deterioration in semen quality. The supplementation of a semen extender with antioxidants can effectively improve sperm quality. Astaxanthin (C_40_H_52_O_4_) is a red carotenoid pigment that provides strong antioxidant properties to improve mitochondrial function to deliver energy (ATP) to sperm and reduce free radicals that prompt harmful effects. However, there have been no previous studies exploring the role of AXT during liquid storage of Hu ram semen at 4 °C. Hence, different concentrations of AXT (Control, 1 µM, 2 µM, 3.5 µM, and 4.5 µM) were added to chilled, stored Hu ram semen to investigate its effect after up to five days of preservation. The results of our research confirm that supplementation of AXT to liquid preservation of Hu ram semen has a significant beneficial effect, and 3.5 µM was revealed to be the optimum concentration for sperm quality.

**Abstract:**

The objective of this research was to investigate the effect of astaxanthin supplementations of semen extender on the quality of Hu ram semen after up to five days of preservation at 4 °C. Semen samples were collected from five healthy Hu rams using an artificial vagina during breeding season (April to August 2023) and diluted with a basic extender supplemented with control (0), 1 µM, 2 µM, 3.5 µM, or 4.5 µM of AXT. Overall, 170 semen ejaculate samples (34 repetitions) from five healthy Hu rams were used in our research study. The results revealed that the addition of AXT (3.5 µM) significantly (*p* ≤ 0.05) increased the sperm kinematic indexes (T.M%, P.M%, MAD%, STR%, and LIN %), sperm viability, plasma membrane integrity, acrosome integrity, total antioxidant content (T-AOC), and mitochondrial membrane potential (MMP) of the Hu rams spermatozoa after up to five days of preservation at 4 °C. Contrary to that, the addition of the best concentration of AXT (3.5 µM) to the semen extender significantly (*p* ≤ 0.05) reduced the reactive oxygen species (ROS) and malondialdehyde (MDA) concentration of Hu ram semen. In conclusion, the results of the current study indicate that the addition of a semen extender with AXT improves the quality of Hu ram spermatozoa by increasing the total antioxidant capacity (T-AOC) and mitochondrial membrane potential (MMP). On the other hand, reducing free radicals induced oxidative (ROS) and per oxidative (MDA) damage to Hu ram semen.

## 1. Introduction

Hu sheep are a medium-sized, dual-purpose (meat and milk) white skin color breed widely distributed in China [1,2]. Hu sheep are famous for their excellent reproductive characteristics, including higher prolificacy, excellent growth rate, and early sexual maturity. Hu sheep are an unseasonal breed (four-season estrous), often experiencing twin and triplet births [3,4]. Natural breeding has shortcomings such as the spread of venereal diseases and transportation of superior-quality male selections, which limit the revelation of higher-potential male animals. Therefore, artificial insemination is a modern technique in reproductive biotechnology that is used for the rapid dissemination of the reproductive potential of higher-quality male animals [5]. Semen conservation is mainly divided into low-temperature preservation (0–5°C), cryopreservation (−196°C), and room-temperature conservation (15–25 °C) [6]. Low-temperature conservation (0–5 °C) has the advantage of stopping the metabolism, growth, and replication of microorganisms. It also improves the quality and survival time of spermatozoa [7].

Semen preservation is mainly affected by extrinsic and intrinsic factors (Temp, pH, osmotic pressure, metabolic rate, etc.) [8,9]. Sperm from almost all animal species contain high concentrations of unsaturated fatty acids in their biological membrane, which make them vulnerable to attack from reactive free-oxygen species, e.g., O_2_^_^ and H_2_O_2_ [10,11]. In mammalian sperm cells, calcium (Ca^2+^) regulates numerous physiological processes, including various enzyme activities, protein phosphorylation, transcription, acrosome reaction, fertilization, and cell death of spermatozoa. Extensive ROS production causes higher (Ca^2+^) uptake into the sperm cell to destroy the sperm plasma membrane combined with the mitochondrial electron transport chain, damaging the sperm nucleus and ultimately leading to apoptosis-related gene activation and cell death of spermatozoa [12,13,14,15,16]. Compared with other domestic animals, ram spermatozoa membrane contains relatively fewer antioxidants, higher polyunsaturated fatty acids (PUFAS), and is more prone to attack by physical, chemical, and free radicals, prompting oxidative damage [17,18]. Consequently, the supplementation of exogenous antioxidants such as AXT constituents to extenders is a proficient method for developing the quality of semen conservation, sperm capacitation, and acrosome reacted sperms and increasing pregnancy rates [19,20,21]. Previous research has shown that the addition of optimum concentrations of antioxidants, melatonin, vitamin E, COQ10, chlorogenic acid, reduced glutathione, etc., has a positive impact on bovine, human, sheep, dog, and rat semen preservation [13,22,23,24,25,26].

Astaxanthin (C_40_H_52_O_4_) is a red carotenoid pigment extracted from alga hematococcus pulvalis, which has antioxidant, anticancer, anti-diabetic, and anti-inflammatory properties. AXT is present in marine organisms (complex plants, shrimps, lobster, microalgae, and salmon) and has recently been utilized in numerous studies [27,28,29]. Modern studies have shown that the addition of astaxanthin improves the motility, viability, and membrane integrity of sperm from rams, bulls, boars, humans, and roosters during preservation [30,31,32,33,34,35]. Astaxanthin has the benefit of easily entering the biological membrane to protect the fatty acid and biological membranes from the harmful effects of lipid peroxidation injuries [36,37]. AXT is a superior antioxidant compared with lutein and β-carotene. The antioxidant properties of AXT are ten times higher than other carotenoids and a hundred times greater than α-tocopherol [29].

However, there are no previous reports on the antioxidant properties of AXT on Hu ram semen preservation at low temperatures (4 °C). Therefore, the main objective of the current study was to investigate the antioxidant capability and potentially protective role of AXT in the Hu ram sperm kinematics index, viability, plasma membrane integrity, acrosome integrity, mitochondrial membrane potential (MMP), total antioxidant capacity (T-AOC), oxidation (ROS), and peroxidation (MDA) status during preservation at 4 °C for up to five days.

## 2. Materials and Methods

### 2.1. Animals and Semen Collection

A total of five healthy Hu rams free from any physical and reproductive ailment (*n* = 5; Age: 4 ± 0.4 years; body condition score (BCS): 3.7 ± 0.4 and body weight (BW): 60 ± 4.5 kg) were raised under a free stall system at the experimental station of Yangzhou University. These rams were fed concentrate containing soybean meal, corn gluten, wheat bran, and canola meal (250–300 gm/animals) twice a day with access to hay and water ad libitum. All experimental techniques performed were approved by the Ethical Committee of Animal Care at Yangzhou University (225009), China, protocol no. SYXK [Su] 2017-0044). This experiment was conducted during the breeding season (April to August) in 2023. For an easy-to-manage workload, the experiment was divided into two parts. The first experiment lasted for three months, from April to June 2023.

During experiment 1, the sperm viability, sperm kinematic index, plasma membrane integrity, and acrosome integrity rate were evaluated. Semen samples were collected twice a week from five healthy Hu rams using the artificial vagina (A.V.) method and taken to the lab at 37 °C within 25 min to avoid denaturation, and initial evaluation was performed for volume, color, concentration, and viability. Only semen ejaculates with volume (0.7–1.8 mL), concentration ≥ 2 × 10^9^/mL, and total motility ≥ 75% were included in this research. Each time (*n* = 5), semen samples were collected and pooled together to prevent individual variation and divided into control (0) and different concentrations (1 µm, 2 µm, 3.5 µm, 4.5 µm) of AXT. Hence, (*n* = 10) samples were collected per week and a total of (*n* = 85) samples were used during the three-month period. During experiment 1, each parameter was repeated (*n* = 9) times to confirm the results and avoid significant variation in the dataset.

The second experiment lasted for two months, from July to August 2023. During experiment 2, the reactive oxygen species (ROS) concentration, malondialdehyde (MDA) concentration, total antioxidant capacity (T-AOC) and mitochondrial membrane potential (MMP) of semen samples were measured using the kits’ instructions. During experiment 2, again, similar procedures were followed for semen collection and evaluation and supplementation of AXT (1 µm, 2 µm, 3.5 µm, 4.5 µm) was performed. Hence, (*n* = 10) samples were collected per week and a total of (*n* = 85) samples were collected during the two-month period. During experiment 2, again, each parameter was repeated (*n* = 9) times to confirm the results and avoid significant variation in the dataset.

### 2.2. Chemicals

Astaxanthin was procured from Solarbio Science and Technology Company, Beijing, China. All other chemicals and reagents used in the experiment were obtained from Sangon Biotech Company, Shanghai, China, unless otherwise specified.

### 2.3. Semen Processing, Supplementation and Preservation

Basic semen extender was prepared by dissolving tris (1.54 g), citric acid (0.82 g), fructose (1.00 g), (25,000 IU), and both procaine penicillin (0.015 g) and streptomycin (0.035 g) in 50 mL of ultrapure distilled water. For protection against low temperature preservation (4 °C) soy lecithin (.05 g) was added to basic diluent by heating at 70 °C. Then, we used 10 mM stock solution of astaxanthin (C_40_H_52_O_4_) purchased from Solarbio Company, Beijing, China, with Catalogue No. A424114 and Lot #K2204151. According to the company’s description, 10 mM stock solution of AXT in DMSO was prepared by dissolving 5 mg (0.005 g) of AXT in 1 mL of DMSO. The resulting stock solution must be protected from direct light and air, stored at −80 °C during transportation, handling and throughout the experiment. We liquefied the deep-frozen and solidified stock solution of AXT at room temperature in dark conditions. Consequently, 1 mL (10 mM) stock solution contained 10,000 µM of AXT in DMSO. Stock solution was divided into small portion of 50 µM by portioning 5 ul into small tubes for subsequent uses. The tubes were protected from light and stored at −80 °C in a deep freezer. Next, 0.5 µL (50 µM) stock solution of AXT in DMSO was further diluted using 1 mL or 1000 µL of basic extender. During the experiment, we made sure that all treatment groups contained an equal volume of DMSO and comprised less than 0.5% solution to prevent their antioxidant effect on sperms. After that, different concentrations of AXT were prepared by adding 20 µL (1 µM), 40 µL (2 µM), 70 µL (3.5 µM), 90 µL (4.5 µM) to semen samples diluted with basic extender 1:9, or the control (0) group without any antioxidant. Hence, the control group contained a 130 µL pooled semen sample diluted with 1170 µL basic extender; the 20 µL (1 µM) group contained a 130 µL pooled semen sample diluted with 1150 µL basic extender + 20 µL (1 µM) AXT solution; the 40 µL (2 µM) group contained a 130 µL pooled semen sample diluted with 1130 µL basic extender + 40 µL (2 µM) AXT solution; the 70 µL (3.5 µM) group contained a 130 µL pooled semen sample diluted with 1100 µL basic extender + 70 µL (3.5 µM) AXT solution; the 90 µL (4.5 µM) group contained a 130 µL pooled semen sample diluted with 1080 µL basic extender +90 µL (4.5 µM) AXT solution.

The sperm kinematics index (total motility TM%, progressive motility P.M%, average motion degree (MAD), straightness (STR) and linearity (LIN)) was assessed every 24 h interval until day 5. Sperm viability, plasma membrane and acrosome integrity were also tested every 24 h until day 5. All other parameters, including sperm reactive oxygen species (ROS), malondialdehyde (MDA), total antioxidant capacity (T-AOC) and mitochondrial membrane potential (MMP), were evaluated on the fifth day of preservation.

### 2.4. Sperm Kinematics Parameters Evaluation

Sperm motility indexes (total motility, progressive motility, average motion degree, straightness and linearity) were measured using an automatic computer-assisted sperm analyzer (CASA) version 5, (Mailang ML-608JZ 11), Nanning, China. Next, 15 µL of semen sample preserved at 4 °C was diluted seven times with basic extender and incubated at 37 °C for a minimum of 3 min. A 1.6 µL drop of incubated semen sample was added to the sperm count slide for evaluation of sperm kinematic characteristics.

### 2.5. Sperm Viability Test

Sperm viability/vitality was evaluated using the eosin-nigrosin staining method. First, a 5 µL diluted semen sample was placed in a test tube. Then, 5 µL of each eosin and nigrosin staining solution was added and mixed well, followed by a 30 s wait. After 30 s, 5 µL of solution was placed on the slide, spread evenly with coverslip and left for 15 min to dry. Observed under 40× microscopic lenses, at least 300 sperm were analyzed to determine the sperm viability/vitality percentage estimation.

### 2.6. Plasma Membrane Integrity Evaluation of Spermatozoa

The plasma membrane integrity of sperm was evaluated using the Hypo osmotic swelling test (HOST) method. First, 100 mL hypo osmotic solution was prepared by mixing 0.90 g fructose and 0.49 g sodium citrate in 100 mL ultrapure distilled water. Next, 15 µL semen samples were mixed in 150 µL HOST solution and incubated for 30 min at 37 °C. At least 200 spermatozoa were counted using 40× phase contrast microscope for swelling and non-swelling of tails to check the percentage of sperm with membrane integrity and loss of integrity.

### 2.7. Acrosome Integrity Evaluation of Spermatozoa

Coomassie brilliant blue G-250 staining was used to evaluate the acrosome integrity of sperms. Coomassie brilliant blue G-250 staining solution was prepared by dissolving (0.10 g) of G-250 dye in 50 mL of 95% ethanol. Then, (100 mL) of 85% phosphoric acid was added and the final volume of staining solution was fixed to 1 L. Briefly, a 50 µL semen sample was placed in a test tube. After 1 mL of 4% paraformaldehyde was added to the fixed semen sample for 10 min, the resulting solution was centrifuged at (2000× *g*) for 5 min to obtain the precipitate of sperm cells. Next, 10 µL of semen sample was spread evenly on a slide with the help of a coverslip to obtain a smear. Finally, coomassie brilliant blue dye (G-250) was used to stain the smear for minimum 30 min. At least 200 sperm heads were evaluated using a 1000× oil immersion lens to determine the percentage of spermatozoa with acrosome integrity and non-integrity.

### 2.8. Assessment of ROS in Semen Samples

A reactive oxygen species assay kit (ROS assay kit Solarbio, Beijing, China) was used for the evaluation of ROS in semen samples. Phosphate-buffered saline (PBS) (500 µL) was used to wash 50 µL of semen samples. Semen samples preserved at 4 °C were treated with 300 µL DCFH-DA working solution and incubated for 30 min at 37 °C. The resulting solution was washed three times with PBS to remove the DCFH-DA from sperm cells and precipitation was obtained via centrifugation at 1500× *g* for 15 min. A multifunctional micro plate reader was used to measure the fluorescence intensity of ROS. The excitation/Emission wavelength of DCF was set to 488/525 nm for measurement of fluorescence intensity of ROS [38].

### 2.9. Assessment of MDA Content of Spermatozoa

The malondialdehyde (MDA) concentration in semen samples was evaluated using a lipid peroxidation assay kit (Beyotime institute of technology, Shanghai, China) and results were obtained through a comparison with a standard curve according to the kit’s instructions. MDA working fluid solution was prepared by mixing three types of reagents: Thiobarbituric acid (TBA) diluent, antioxidant and TBA formulations. Briefly, 200 µL semen samples were taken from each test tube and centrifuged at 200 rpm/10 min to obtain the supernatant. MDA working solution (220 µL) was added to 110 µL of supernatant, and the resulting solution was placed in a dark and hot water bath at 100 °C for 15 min. After 15 min, it was removed from the hot water bath and left to rest for 5 min to cool down, then centrifuged again at 2500 rmp/10 min. Next, 100 µL of each three replicate was placed in a 96-well plate and samples were observed at 532 nm wavelength using a microporous multifunctional fluorescence detection system. The MDA concentration was assessed as nmol/mL of protein [39].

### 2.10. Assessment of T-AOC Content of Spermatozoa

The total antioxidant capacity of semen samples was measured using a (T-AOC) assay kit (Nanjing Institute of Biotechnology, Nanjing, China). The standard curve was obtained using dilution of standard Trolox solution (10 mM) with distilled water to 0.1, 0.2, 0.4, 0.8 and 1 mM. Semen precipitations were obtained via centrifugation of 100 µL of semen sample at 2000 rpm/10 min. After centrifugation, 10 µL of supernatant was added to each hole of a 96-well plate. Afterward, 20 µL of enzyme solutions and 170 µL of ABTS working solution were added and mixed well according to the kit’s instructions. The resulting mixture was allowed to react at room temperature for a minimum of 6 min and observed at 405 nm wavelength using a fluorescence multifunctional detection probe. Results were obtained using a standard curve and expressed as nmol/L of protein [40].

### 2.11. Assessment of Mitochondrial Membrane Potential of Spermatozoa

A mitochondrial membrane potential (MMP) assay kit (Beyotime Institute of Technology, Shanghai, China) was used to measure the mitochondrial membrane potential of the spermatozoa. When (MMP) was higher, JC-1 accumulated in the mitochondrial matrix, developing polymer and generating red-color fluorescence. When (MMP) was lower, JC-1 failed to gather in the mitochondrial matrix. In its resting state, it became a monomer and created green fluorescence. Semen samples were washed twice with phosphate-buffered saline (PBS); then the supernatant was discarded via centrifugation at 2000 rmp for 10 min, and JC-1 working solution dye (500 µL) was added and incubated in darkness at 37 °C for 30 min according to the kit’s guidelines. After incubation, the samples were washed three times with JC-1 staining solution. Fluorescence intensity ratio of red (Excitation/Emission = 525/590) and green fluorescence (Excitation/Emission = 488/525) was measured using a multifunctional plate reader and often used to evaluate the degree of mitochondrial membrane potential (MMP) [41].

### 2.12. Statistical Analysis

All the datasets collected during the experiment were statistically examined using Social Science Packages (SPSS, IBM, version 24). To check the normal distribution of data, the Shapiro–Wilk test was used, which displayed data as normally distributed. All the results were displayed as mean ± SEM and statistical significance was set at (*p* ≤ 0.05). The mean values of sperm motility index %, sperm viability, plasma membrane integrity, acrosome integrity, ROS concentration, MDA level, total antioxidant content (T-AOC) and mitochondrial potential (MMP) were evaluated using a one-way ANOVA test, and mean values were compared using Duncan’s multiple range test procedure.

## 3. Results

### 3.1. Effect of AXT Supplementation on Sperm Viability

The results showed that the sperm viability of 3.5 µM group was statistically higher (*p* < 0.5) than the control and the 1 µM group on the first day of preservation. The sperm viability of the 2 µM, 3.5 µM and 4.5 µM groups was statistically higher (*p* < 0.5) than that of the control and 1 µM groups on the second and fifth day. The sperm viability of the 3.5 µM group was statistically higher (*p* < 0.5) than that of the control group on the third day. The sperm viability of the control group was significantly lower (*p* > 0.5) than that of all AXT supplementation groups on the fourth day of storage, as shown in Table 1.

### 3.2. Effect of AXT Supplementation on Sperm Kinematic Index

The total motility % of 2 µM, 3.5 µM and 4.5 µM groups was statistically higher (*p* < 0.5) than that of the control and 1 µM groups on the first and fourth day of preservation. The sperm TM % of 3.5 µM and 4.5 µM groups was statistically higher (*p* < 0.5) than that of the control and other groups on the second and third day of storage. The sperm TM% of the control group was statistically lower (*p* > 0.5) than that of all AXT supplementation groups on the fifth day of preservation at 4 °C, as shown in Table 1.

The sperm progressive motility % of 2 µM, 3.5 µM and 4.5 µM groups was statistically higher (*p* < 0.5) than that of the control and 1 µM groups on the first day of preservation. The sperm PM% of the 3.5 µM and 4.5 µM groups was statistically higher (*p* < 0.5) than that of the control and other groups on the second and third day of storage. The sperm PM% of the control group was statistically lower (*p* > 0.5) than that of all AXT supplementation groups on the fourth and fifth day of preservation at 4 °C, as shown in Table 1.

The sperm average motion degree (MAD) of all AXT supplementation groups was statistically higher (*p* < 0.5) than that of the control group on the second day of preservation. The sperm MAD of the 3.5 µM and 4.5 µM groups was statistically higher (*p* < 0.5) than that of the control and other groups on the third day of storage. The sperm MAD of the 3.5 µM group was statistically higher (*p* < 0.5) than that of the control and other groups on the fifth day of storage, as shown in Table 1.

There was no significant difference in the linearity % of the control and all AXT supplementation groups on the first day of preservation. The sperm LIN % of all AXT supplementation groups was statistically higher (*p* < 0.5) than that of the control group on the fifth day of preservation at 4 °C. Similarly, there was no significant difference in straightness % of control and all AXT supplementation groups on the first day of preservation. The sperm STR % of the control group was statistically lower (*p* > 0.5) than that of all AXT supplementation groups on the fifth day of preservation, as shown in Table 1.

### 3.3. Effect of AXT Supplementation on Sperm Plasma Membrane Integrity

On the first day of preservation, the plasma membrane integrity of the 2 µM and 3.5 µM groups was statistically higher (*p* < 0.5) than that of the control and 1 µM groups. The plasma membrane integrity of the 3.5 µM and the 4.5 µM groups was statistically higher (*p* < 0.5) than that of the control and 1 µM groups on the second day. The plasma membrane integrity of the control group was statistically lower (*p* > 0.5) than that of all AXT supplementation groups on the third day. The plasma membrane integrity of the 2 µM, 3.5 µM and 4.5 µM groups was statistically higher (*p* < 0.5) than that of the control and 1 µM groups on the fourth and fifth days of preservation at 4 °C, as shown in Table 2.

### 3.4. Effect of AXT Supplementation on Sperm Acrosome Integrity

On the first day of preservation, the acrosome integrity of 2 µM and 3.5 µM groups was statistically higher (*p* < 0.5) than that of the control and 1 µM groups. The acrosome integrity of the 2 µM and 3.5 µM groups was statistically higher (*p* < 0.5) than that of the control group on the second day. The acrosome integrity of the control group was statistically lower (*p* > 0.5) than that of all AXT supplementation groups on the third and fourth day. The acrosome integrity of the 2 µM, 3.5 µM and 4.5 µM groups was statistically higher (*p* < 0.5) than that of the control group on the fifth day of preservation at 4 °C, as shown in Table 2.

### 3.5. Effects of AXT Supplementations on ROS Content of Hu Ram Spermatozoa

**Figure 1:**
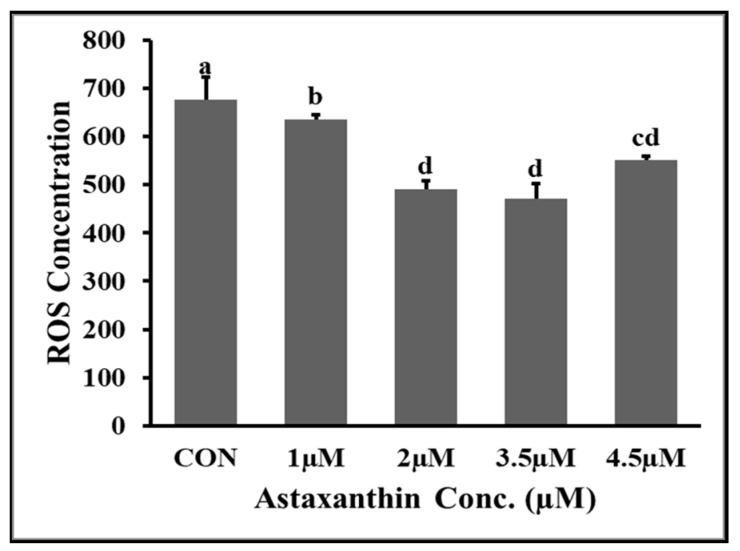
Effect of different concentrations of AXT on ROS concentration of Hu ram spermatozoa stored at 4 °C on 5th day. Note: (*p* ≤ 0.05) indicates a significant difference which is shown by different letters above the columns, but (*p* ≥ 0.05) indicates a non-significant difference shown by the same letters above the columns.

The effects of different concentrations of astaxanthin on reactive oxygen species (ROS) content of Hu ram spermatozoa stored at 4 °C on the fifth day of preservation are shown in Figure 1. The results showed that the ROS content of the 3.5 µM and 2 µM groups was significantly lower (*p* ≤ 0.05) than that of the control and 1 µM groups. The ROS level of the 2 µM and 3.5 µM groups was non-significantly (*p* ≥ 0.05) lower than that of the 4.5 µM group. The ROS content of the 1 µM group was significantly (*p* ≤ 0.05) lower than that of the control group.

### 3.6. Effects of Astaxanthin Supplementations on MDA Content of Hu Ram Spermatozoa

**Figure 2:**
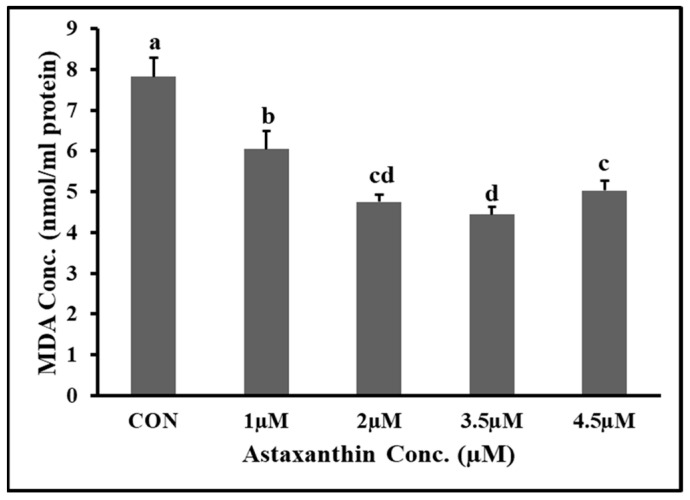
Effect of different concentrations of AXT on MDA content of Hu ram spermatozoa stored at 4 °C on 5th day of preservation. Note: (*p* ≤ 0.05) indicates significant difference, which is shown by different letters above the column, while (*p* ≥ 0.05) indicates non-significant difference shown by the same letters above the column.

The effects of different concentrations of AXT on the malondialdehyde (MDA) content of Hu ram spermatozoa stored at 4 °C on the fifth day of preservation are shown in Figure 2. The results showed that the MDA content of the 3.5 µM and 2 µM groups was significantly lower (*p* ≤ 0.05) than that of the control and 1 µM groups. The MDA content of the 2 µM group was non-significantly (*p* ≥ 0.05) lower than that of the 4.5 µM group. The MDA content of the 1 µM group was significantly (*p* ≤ 0.05) lower than that of the control group.

### 3.7. Effect of AXT on T-AOC Capacity of Hu Rams Spermatozoa

**Figure 3:**
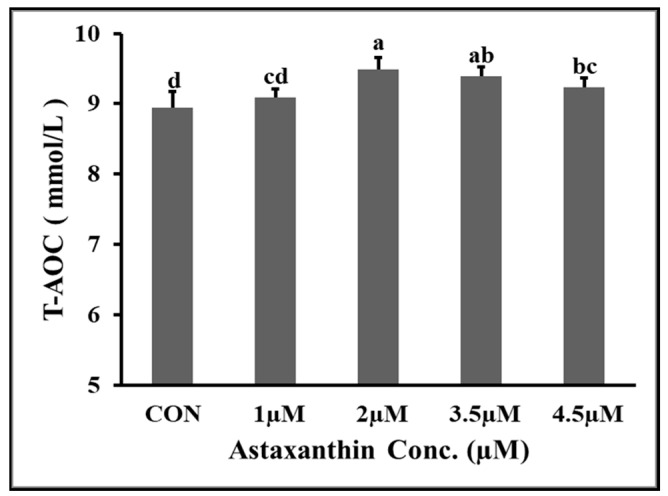
Effect of different concentrations of AXT on (T-AOC) of Hu ram spermatozoa preserved at 4 °C on 5th day of storage. Note: (*p* ≤ 0.05) indicates significant difference among the groups, which is shown by different letters above the columns. However, (*p* ≥ 0.05), indicates non-significant difference among the groups which is shown by the same letters above the columns in the figure.

The effects of different concentrations of AXT on the total antioxidant capacity of Hu ram spermatozoa stored at 4 °C on the fifth day of preservation are shown in Figure 3. The results show that the (T-AOC) of the 2 µM and 3.5 µM groups was significantly higher (*p* ≤ 0.05) than that of the control and 1 µM groups. The T-AOC of the 3.5 µM group was non-significantly (*p* ≥ 0.05) higher than that of the 4.5 µM group. The T-AOC of the 1 µM group was non-significantly (*p* ≥ 0.05) higher than that of the control group.

### 3.8. Effects of AXT on Mitochondrial Membrane Potential (MMP) of Hu Ram Spermatozoa

**Figure 4:**
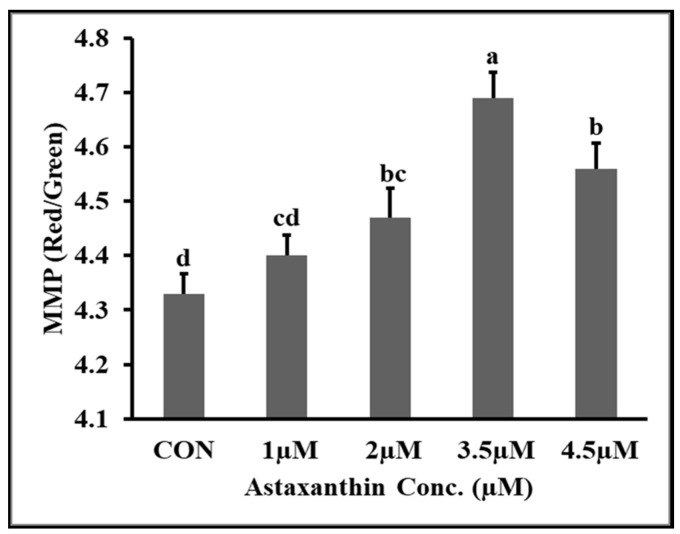
Effect of different concentrations of AXT on MMP of Hu ram spermatozoa stored at 4 °C on 5th day. Note: (*p* ≤ 0.05) indicates significant difference, which is shown by different letters above the columns, while (*p* ≥ 0.05) indicates non-significant difference shown by same letters above the columns.

The effects of different concentrations of AXT on the mitochondrial membrane potential (MMP) of Hu ram spermatozoa stored at 4 °C on the fifth day of preservation are shown in Figure 4. The results shows that the MMP of 3.5 µM groups was significantly higher (*p* ≤ 0.05) than that of the control and all other supplementation groups. The mitochondrial potential of the 4.5 µM group was significantly higher (*p* ≤ 0.05) than that of the control and 1 µM groups. The mitochondrial membrane potential (MMP) of the control group was significantly lower (*p* ≥.05) than that of all the AXT supplementation groups.

## 4. Discussion

The results of our experiment show that supplementation of AXT to basic extender considerably increased the sperm kinematic index (T.M%, P.M%, MAD, STR, LIN), livability, membrane integrity, T-AOC, and MMP and significantly decreased the oxidative stress (ROS) and malondialdehyde (MDA) concentration in Hu ram semen for up to five days of preservation at 4 °C. Similarly, supplementation of AXT to human and rat semen not only significantly increased the normal spermatozoa, longevity, straight-line movement, and fertility potential, but also significantly reduced ROS production [42,43]. Dietary supplementation of AXT to rats with calorie restriction increased the total sperm count, motility, and total antioxidant capacity and ameliorated infertility in male rats [44]. A balance between excessive ROS production during metabolism of motile sperm cells and their inactivation by the antioxidant defense system is required to maintain normal cell function [21].

Reduced oxidative damages due to decreased ROS production and MDA concentration in our study might be due to the lipophilic properties of AXT, which enable them to easily infiltrate the biological membrane by devising effective antioxidant features [45,46]. The higher sperm kinematic index was caused by the positive effect of AXT on the mitochondrial membrane, which allowed it to provide enough energy (ATP) for normal cell functioning and reduced free-radical production inside the cells [47].

Compatible with another study on mice sperm viability, normal morphology, motility index, and antioxidant enzyme activities (T-AOC) were higher in our study due to CatSper1 and CatSper2 gene expression. Conversely, DNA damage and oxidative stress induced by lipid peroxidation (MDA) was significantly lower in our study [48]. Another study in mice demonstrated that AXT significantly restored sperm DNA damage caused by cyclophosphamide testicular toxicity confirmed by histological examination [20].

Moreover, supplementation of AXT to capacitation buffer induced structural and morphological alteration in human sperm cell membrane to increase capacitation, acrosome reaction cell (ARC), Lyn translocation to head region, and ultimately oocyte fertilizing ability in males with idiopathic infertility [34,37]. Our findings in Hu sheep were congruent with dietary supplementation of AXT in arctic char (coldwater fish) and goldfish that improved semen quality, sperm concentration, and fertilization rate due to negative correlation between antioxidant enzyme activity and lipid peroxidation [49,50]. Naturally, various antioxidant enzyme (CAT, SOD, and GPX) defense systems are present inside sperm cells to protect them against oxidative damage [51]. Consistent with our findings, dietary intake of astaxanthin nanoparticles (AXT-NPs) in roosters improved sperm cryosurvival, antioxidant enzyme activity, catalase (CAT), superoxide dismutase (SOD), and glutathione peroxidase (GPX). It also increased the total antioxidant capacity (TAOC) and reduced the lipid peroxidation (MDA) concentration by alleviating damages caused by heavy metal cadmium [35].

Many types of antioxidant have been tested previously to reduce or counter free radical-induced oxidative damage in various avian and mammalians species [52,53,54,55,56]. The addition of optimum concentrations of AXT to bull, rooster, human, boar, rat and also Hu ram semen in our experiment significantly improved the sperm motility index, viability, plasma membrane integrity, acrosome integrity, mitochondrial membrane potential, and total antioxidant content [31,32,33,35,36,37,42,43]. In previous studies, 2 µM and 4 µM proved to be the most effective concentrations of AXT for cryopreservation (−196 °C) of Moghani rams and low-temperature (4 °C) preservation of Han ram spermatozoa up to 72 h [30,57]. A similar protective role of AXT in anti-tumor activity that reduced oxidative stress and promoted mitochondrial functions was observed previously [58].

Addition of AXT to Han rams semen (4 °C) storage and Moghani ram semen cryopreservation (−196 °C) profoundly improved sperm variables and reduced oxidative damages (ROS and MDA concentration), which was consistent with our findings in Hu ram semen preservation. However, in our study, 3.5 µM was found to be the optimum concentration of AXT for Hu ram semen preservation at (4 °C) for up to five days. Supplementation with 1 µM and 2 µM AXT might not be sufficient to fully activate the antioxidant enzymes’ defense system and mitochondrial electron transport chain complex to provide energy for ideal sperm cell functioning. Extra supplementation 4.5 µM or above probably has some harmful effect on sperm variables and biochemical metabolites. Furthermore, breeds, season, nutrition and differentially expressed protein association with the preservation process also affect the optimum supplementation of antioxidants. There are no previously published studies describing the effects of AXT supplementation on average motion degree (MAD), straightness (STR) and linearity (LIN) percentage as well as mitochondrial membrane potential (MMP) and total antioxidant capacity (T-AOC) of ram semen for up to five days of preservation at (4 °C).

## 5. Conclusions

In conclusion, this is a major study describing the addition of AXT to Hu ram semen storage at (4 °C) for up to five days of preservation. Supplementation of the optimum concentration of AXT (3.5 µM) to Hu ram semen preservation significantly improves its kinematic properties, longevity, plasma membrane integrity, acrosome integrity, total antioxidant content, and mitochondrial membrane potential. On the other hand, addition of AXT (3.5 µM) significantly reduces reactive oxygen species (ROS) and malondialdehyde (MDA) concentrations of spermatozoa to improve their longevity and, ultimately, their fertilizing ability.

## Figures and Tables

**Table 1:** Effects of different concentrations of astaxanthin (AXT) on kinematic parameters of Hu ram spermatozoa stored at 4 °C.

Index (%)	Time (d)	Control	1 µM	2 µM	3.5 µM	4.5 µM
Viability (%)	0	81.87 ± 1.7	82.12 ± 1.09	82.09 ± 1.1	82.77 ± 0.9	82.05 ± 0.76
	1	67.89 ± 1.05 ^b^	68.08 ± 1.19 ^b^	72.74 ± 0.63 ^ab^	76.91 ± 0.11 ^a^	72.94 ± 1.46 ^ab^
	2	63.52 ± 0.46 ^c^	62.55 ± 1.41 ^c^	65.68 ± 0.82 ^b^	71.19 ± 0.98 ^a^	69.47 ± 0.74 ^ab^
	3	57.21 ± 1.86 ^b^	59.98 ± 2.76 ^ab^	62.01 ± 0.45 ^ab^	63.82 ± 0.75 ^a^	61.59 ± 1.05 ^ab^
	4	51.66 ± 0.95 ^c^	53.95 ± 0.93 ^b^	57.71 ± 0.20 ^a^	58.4 ± 0.58 ^a^	56.64 ± 0.56 ^ab^
	5	42.49 ± 1.43 ^c^	44.62 ± 0.97 ^c^	50.27 ± 0.93 ^b^	53.59 ± 0.53 ^a^	49.88 ± 0.93 ^b^
T.M (%)	0	79.72 ± 0.86	78.98 ± 1.32	81.23 ± 0.63	82.10 ± 1.21	80.75 ± 0.55
	1	64.24 ± 1.44 ^c^	65.21 ± 1.74 ^c^	70.5 ± 0.22 ^b^	75.99 ± 1.52 ^a^	70.77 ± 0.71 ^b^
	2	62.48 ± 0.38 ^b^	61.65 ± 0.66 ^b^	62.7 ± 0.57 ^b^	70.48 ± 0.88 ^a^	69.05 ± 1.04 ^a^
	3	52.13 ± 0.64 ^b^	53.79 ± 1.18 ^b^	53.99 ± 1.23 ^b^	62.57 ± 0.84 ^a^	60.98 ± 0.92 ^a^
	4	42.57 ± 0.71 ^b^	43.86 ± 1.33 ^b^	48.97 ± 0.57 ^a^	50.46 ± 2.19 ^a^	50.14 ± 1.8 ^a^
	5	29.1 ± 1.47 ^d^	35.27 ± 0.56 ^c^	38.44 ± 0.34 ^b^	45.24 ± 1 ^a^	37.27 ± 0.97 ^bc^
P.M (%)	0	73.58 ± 0.32	71.67 ± 0.76	74.50 ± 1.10	75.43 ± 1.17	73.43 ± 0.91
	1	56.31 ± 1.25 ^b^	57.49 ± 1.96 ^b^	63.56 ± 1.02 ^a^	67.91 ± 1.95 ^a^	62.84 ± 1.45 ^a^
	2	53.26 ± 0.93 ^b^	53.48 ± 0.89 ^b^	55.8 ± 0.7 ^b^	62.18 ± 1.14 ^a^	60.66 ± 0.59 ^a^
	3	45.54 ± 1.47 ^b^	48.18 ± 0.95 ^b^	46.79 ± 1.17 ^b^	53.9 ± 1.94 ^a^	54.01 ± 1.21 ^a^
	4	34.1 ± 0.44 ^c^	36.22 ± 1.57 ^bc^	39.46 ± 0.83 ^ab^	42.62 ± 0.94 ^a^	43.42 ± 1.96 ^a^
	5	21.83 ± 0.62 ^d^	28.69 ± 0.61 ^c^	30.84 ± 0.21 ^b^	36.7 ± 0.42 ^a^	30.21 ± 0.97 ^bc^
MAD (%)	0	57.43 ± 1.30	56.34 ± 1.02	58.31 ± 0.54	60.22 ± 2.31	59.41 ± 2.01
	1	52.82 ± 0.66	43.35 ± 1.67	51.61 ± 6.56	58.25 ± 7.95	51.06 ± 2.45
	2	37.57 ± 1.43 ^c^	38.66 ± 2.21 ^ab^	43.82 ± 3.44 ^ab^	49.98 ± 3.96 ^a^	49.1 ± 4.75 ^a^
	3	35.39 ± 1.61 ^b^	36.44 ± 0.81 ^b^	34.51 ± 2.85 ^b^	48.16 ± 3.91 ^a^	47.2 ± 2.91 ^a^
	4	29.69 ± 2.98 ^ab^	24.27 ± 1.63 ^b^	32.04 ± 3.35 ^ab^	32.66 ± 0.43 ^ab^	35.56 ± 3.12 ^a^
	5	16.72 ± 1.09 ^b^	24.14 ± 3 ^b^	25.86 ± 2.96 ^ab^	33.9 ± 2.38 ^a^	24.14 ± 3.93 ^b^
LIN (%)	0	0.61 ± 0.03	0.60 ± 0.01	0.59 ± 0.02	0.59 ± 0.01	0.58 ± 0.02
	1	0.59 ± 0.01 ^a^	0.58 ± 0.01 ^a^	0.57 ± 0.01 ^ab^	0.58 ± 0.01 ^a^	0.57 ± 0.01 ^ab^
	2	0.56 ± 0.01	0.55 ± 0.01	0.56 ± 0	0.55 ± 0.01	0.54 ± 0.01
	3	0.53 ± 0.02	0.53 ± 0.01	0.54 ± 0.01	0.53 ± 0.01	0.52 ± 0.01
	4	0.53 ± 0.02	0.54 ± 0.01	0.53 ± 0.01	0.53 ± 0.01	0.52 ± 0.02
	5	0.49 ± 0.01 ^c^	0.54 ± 0.02 ^a^	0.52 ± 0.01 ^ab^	0.53 ± 0.01 ^ab^	0.54 ± 0.01 ^a^
STR (%)	0	0.87 ± 0.03	0.86 ± 0.02	0.85 ± 0.01	0.86 ± 0.04	0.85 ± 0.03
	1	0.84 ± 0.01 ^a^	0.83 ± 0.01 ^a^	0.8 ± 0.02 ^ab^	0.83 ± 0.01 ^a^	0.81 ± 0.01 ^ab^
	2	0.79 ± 0.02	0.78 ± 0.01	0.8 ± 0.01	0.78 ± 0.02	0.77 ± 0.01
	3	0.74 ± 0.03	0.75 ± 0.01	0.76 ± 0.02	0.75 ± 0.01	0.73 ± 0.02
	4	0.76 ± 0.04	0.76 ± 0.02	0.75 ± 0.02	0.75 ± 0.01	0.74 ± 0.03
	5	0.67 ± 0.01 ^c^	0.75 ± 0.02 ^a^	0.73 ± 0.02 ^ab^	0.74 ± 0.01 ^ab^	0.76 ± 0.02 ^a^

Note: Duncan’s test was used to check the statistical significant among various groups. If (*p* ≤ 0.05), it represents significant difference among various groups within the rows indicated by different letters. If (*p* ≥ 0.05), it means there is no significant difference among various groups within the rows indicated by the same letters.

**Table 2:** Effects of different concentrations of astaxanthin (AXT) on plasma membrane and acrosome integrity of Hu ram spermatozoa stored at 4 °C.

Index (%)	Time (d)	Control	1 µM	2 µM	3.5 µM	4.5 µM
Plasma membrane integrity	0	61.34 ± 1.27	62.58 ± 0.96	62.48 ± 1.46	63.92 ± 1.72	62.68 ± 1.37
	1	50.63 ± 1.68 ^b^	51.1 ± 1.96 ^b^	57.90 ± 1.94 ^a^	59.73 ± 1.69 ^a^	56.44 ± 1.76 ^ab^
	2	40.99 ± 1.45 ^b^	40.45 ± 1.15 ^b^	44.65 ± 1.35 ^ab^	47.07 ± 1.01 ^a^	46.98 ± 1.07 ^a^
	3	32.54 ± 1.27 ^c^	39.68 ± 0.86 ^b^	41.48 ± 1.86 ^ab^	44.92 ± 1.92 ^a^	41.68 ± 1.27 ^ab^
	4	29.67 ± 0.33 ^b^	29.92 ± 0.72 ^b^	36.79 ± 2.17 ^a^	37.93 ± 1.40 ^a^	34.13 ± 1.06 ^a^
	5	22.76 ± 0.89 ^c^	25.20 ± 1.08 ^c^	33.36 ± 0.61 ^a^	33.15 ± 1.29 ^a^	29.02 ± 1.54 ^b^
Acrosome Integrity	0	91.2 ± 0.76	90.67 ± 0.45	91.66 ± 0.37	91.56 ± 0.85	90.89 ± 0.65
	1	84.12 ± 0.84 ^c^	85.1 ± 0.33 ^bc^	87.4 ± 0.42 ^a^	88.11 ± 0.43 ^a^	86.14 ± 0.83 ^ab^
	2	83.31 ± 0.35 ^b^	84.87 ± 1.07 ^ab^	86.03 ± 0.49 ^a^	86.3 ± 0.82 ^a^	85.46 ± 0.42 ^ab^
	3	81.51 ± 1.13 ^b^	84 ± 0.45 ^a^	84.32 ± 0.12 ^a^	85.47 ± 0.77 ^a^	84.94 ± 0.41 ^a^
	4	80.29 ± 0.68 ^b^	82.06 ± 0.30 ^a^	83.18 ± 0.56 ^a^	83.48 ± 0.21 ^a^	81.88 ± 0.64 ^a^
	5	74.69 ± 0.52 ^b^	76.93 ± 1.08 ^ab^	79.53 ± 1.24 ^a^	79.8 ± 0.57 ^a^	78.91 ± 0.69 ^a^

Note: Duncan’s test was used to check the statistical significance among various groups. If (*p* ≤ 0.05), it means there is a significant difference among various groups within the rows indicated by different letters. If (*p* ≥ 0.05), it means there is no significant difference among various groups within the rows indicated by the same letters.

## Data Availability

All datasets collected, utilized and statistically evaluated during the present research are accessible from the authors upon reasonable request. The data are not publicly available due to some confidential agreement.
